# Confidence in incomplete visual search

**DOI:** 10.1080/13506285.2025.2571626

**Published:** 2025-10-30

**Authors:** Hui Men, Alexander C. Schütz

**Affiliations:** a Allgemeine und Biologische Psychologie, Philipps-Universität Marburg, Marburg, Germany; b Center for Mind, Brain and Behaviour, Philipps-Universität Marburg, Marburg, Germany

**Keywords:** Visual search, eye movements, metacognition, confidence

## Abstract

Visual search is critical in many activities. Under time pressure or when targets are hard to detect, decisions about target presence might be forced with insufficient information. Then an estimation of confidence is essential for subsequent actions. We asked participants to search for a small target in two search stimuli and to choose the one they felt more confident about. Target presence and the size of one stimulus was varied. The hit rate decreased as gaze-target distance increased. At large distances, hits and false alarms were similar and correlated across participants, suggesting lucky guesses. Performance was better in chosen than in non-chosen stimuli, mainly because of higher hit rate. Confidence choices preferred smaller stimuli and target-present responses. The latter preference decreased with increasing gaze-target distance, indicating reliance on target visibility. These results indicate that confidence in visual search is influenced by target visibility and the probability that the target was overlooked.

Visual search is a ubiquitous task (for reviews see Eckstein, 2011; Wolfe, 2010) in many everyday activities (for instance, searching for keys in a room or a friend in a crowd) and is also a crucial skill in many professions (for instance, airport security screening (e.g., Biggs et al., 2013) or radiology (for a review see van der Gijp et al., 2017)). Although it might appear like a simple task, it involves many facets of sensorimotor control: visual aspects, such as the visibility of the target at different eccentricities and the sampling of information with eye movements (e.g., Najemnik & Geisler, 2005); cognitive aspects, such as prior knowledge about the most likely target location (e.g., Geng & Behrmann, 2005; Paeye et al., 2016) or the prevalence of the target (e.g., Wolfe et al., 2005, 2007); and metacognitive aspects, such as the decision when to terminate the search (e.g., Cain et al., 2012; Chun & Wolfe, 1996). Here, we focus on the metacognitive estimation of confidence after search has been terminated prematurely.

Often, visual search is terminated by the searcher, either when the target was found or when sufficient evidence has been gathered that the target is not present. Under these conditions, reaction times are typically shorter in target-present than in targetabsent trials (Wolfe et al., 2005). Furthermore, decisions about when to terminate search depend on expectations about the prevalence of the target (Wolfe et al., 2005) or the number of available targets (Cain et al., 2012). Interestingly, under extreme conditions of low target prevalence (Wolfe et al., 2005) or large 3D search spaces (Lago et al., 2021), search is terminated too early, leading to frequent misses. Interestingly, some of the misses of rare targets can be corrected with longer deliberation time (Fleck & Mitroff, 2007), suggesting that the target-absent responses were indeed reached too quickly.

Search can also be terminated prematurely due to external factors, for instance, time restrictions or interfering events. In these cases, searchers might be forced to decide about the target's presence, not only before they have found it, but also before they have scanned the search area sufficiently. Then it is important for searchers to be able to estimate how confident they are about their decisions in order to plan subsequent actions accordingly. This metacognitive ability presumably requires knowledge about the visibility of the target at different retinal eccentricities, the cumulative area that was scanned during the search and the prevalence of the target.

Metacognitive abilities allow humans to estimate the quality of their perceptual decisions (for reviews see Mamassian, 2016, 2020; Schulz et al., 2023; Yeung & Summerfield, 2012). Subjective confidence typically reflects objective performance and thereby takes into account internal uncertainty (Barthelme & Mamassian, 2009) and not just stimulus properties as proxies (Barthelme & Mamassian, 2010). Furthermore, subjective confidence affects the decision about whether to seek additional information to improve the perceptual judgment (Desender et al., 2018, 2019). Nevertheless, subjective confidence can be dissociated from objective performance (for a review see Shekhar & Rahnev, 2021), sometimes with large discrepancies, for example overconfidence for perceptually completed percepts (Baykan et al., 2025; Ehinger et al., 2017; Gloriani & Schütz, 2019) or underconfidence in blindsight (Sahraie et al., 1998).

Most importantly in the context of this study, humans can estimate the difficulty of visual search, for instance when the number of distractors or the difference between distractors and targets is varied (Godwin & Hout, 2023; Miller et al., 1985; Redford et al., 2011; Reyes & Sackur, 2014).

Here, we investigated if searchers have metacognitive insight to improve their search decisions and how searchers determine their confidence when the duration of search is limited and a premature decision about the presence of the target is forced.

## Methods

### Design

**Figure 1:**
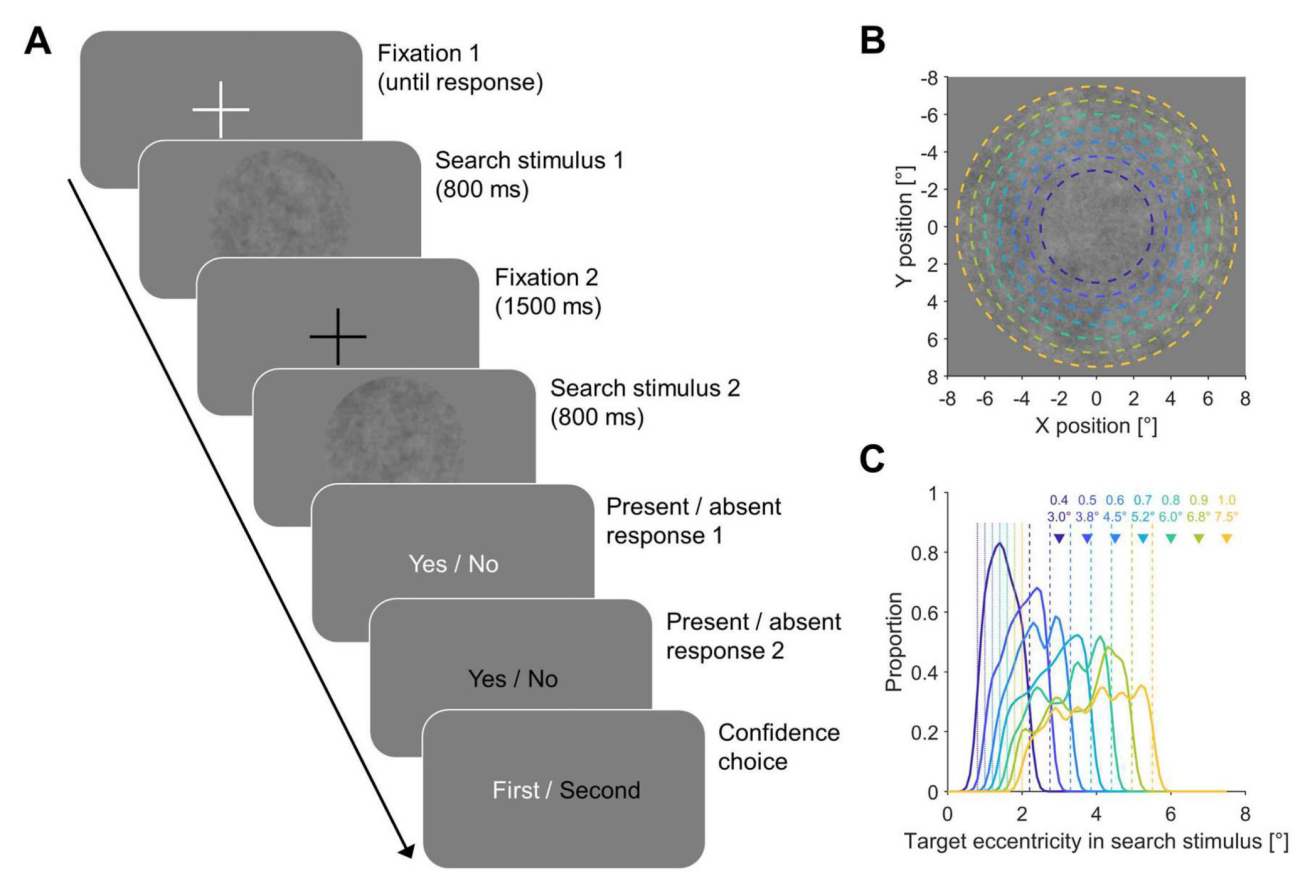
Experimental paradigm and stimuli. (A) Trial procedure. Participants saw two search stimuli and then had to decide if the target was present or absent in each of the stimuli and for which of the two stimuli they were more confident. (B) Search area with different scales. The search area was a 1/f noise with an RMS contrast of 0.07. Coloured dashed lines denote the size of the search area at different scales. (C) Distributions of target eccentricities in the search stimulus for different scales. Triangles denote the size of the search area at different scales. Dotted and dashed lines denote the minimum and maximum eccentricity of targets, respectively.

To study confidence in visual search, we used a confidence forced-choice paradigm (Mamassian, 2020). In each trial, participants had to search for a small target in two subsequent search stimuli. At the end of each trial, they first had to report the presence or absence of the target for each search stimulus and then to choose for which stimulus they felt more confident (Figure 1A). This confidence forced-choice paradigm has two advantages compared to confidence ratings: first, it does not require an arbitrary and subjective mapping between internal confidence and an external scale and second, it allows to directly compare confidence for pairs of stimuli. We found that search performance was highly correlated in the two intervals and that confidence was not biased towards one of the two intervals (Supplementary material, Figure S1), indicating that the paradigm produces highly reliable and unbiased data.

The size of the search area was varied in seven steps (Figure 1B). The scale of the comparison stimulus was 0.4, 0.5, 0.6, 0.7, 0.8, 0.9, and 1. The scale of the standard stimulus was always 0.7. For each scale, a target could be present only in the standard stimulus (1-0), only in the comparison stimulus (0-1), in both stimuli (1-1) or in neither of the two stimuli (0-0). The order of the standard and comparison stimulus was counterbalanced across trials. Each condition was repeated 10 times, resulting in a total of 280 trials (7 scales × 4 conditions × 10 repetitions). Additionally, 12 practice trials were conducted ahead, which were not included in the analysis.

### Participants

Twenty-four students from the University of Marburg (18 women, 6 men, mean age = 23 years, range = 18–33 years) participated in the experiment. All participants had normal or corrected-to-normal vision and provided written informed consent. All participants were naïve to the purpose of the experiment. They could choose if they wanted to be compensated with course credits or monetarily with 8€/h for the participation of the experiment. The experiments were conducted in accordance with the Declaration of Helsinki (1964) and approved by the local ethics committee of the Psychology Department at Marburg University (proposal number 2021-71k). The data of two participants were excluded because more than 10% of trials were deemed invalid (see below).

### Apparatus and stimuli

The stimuli were displayed using the Psychtoolbox (Brainard, 1997; Kleiner et al., 2007; Pelli, 1997) in MATLAB R2017b. Participants sat in front of a VIEW-Pixx monitor (VPixx Technologies Inc., Saint-Bruno, QC, Canada) at a viewing distance of 60 cm. The size of the monitor was 51.50 × 29.00 cm, with a spatial resolution of 1,920 × 1,080 pixels and a refresh rate of 120 Hz. The monitor output was linearized with luminance values of 0.39, 54, and 105 cd/m^2^ for black, grey and white. The background of the display was grey (R = G = B = 128). Controlled by the Eyelink Toolbox (Cornelissen et al., 2002), eye movements of the right eye were recorded using an EyeLink 1000+ (SR Research Ltd., ON, Canada) at a sampling rate of 1,000 Hz. The eye tracker was calibrated before the main experiment and re-calibrated after 150 trials.

The fixation cross and the search area were shown in the centre of the screen. The search area was a 1/f noise with an RMS contrast of 0.07. Presented as a circle, the radius of the search area was a scale of 7.5° (scale × 7.5°). The scale of the standard stimulus was fixed to 0.7. The scale of the comparison stimulus varied from 0.4 to 1 with a step size of 0.1 (0.4 × 7.5°, 0.5×7.5°, …, 1×7.5°), resulting in 7 sizes of search area (Figure 1B).

The target was a Gabor patch with a spatial frequency of 10 c/° oriented at an angle of 45°. The standard deviation of the Gaussian envelope was 0.1°. The position of the target was randomized within a radius between scale ×2° and scale ×5.5°.

The contrast of the target was adjusted for each participant individually in a pre-test. In this pre-test, each trial contained one stimulus, with the target presented either on the left or the right of the fixation location at an eccentricity of 1.2°. The search area had a fixed scale of 0.4 and disappeared after 800 ms, after which participants had to determine on which side of the search area the target was presented (Left/Right) by pressing “F” (Left) or “J” (Right). There was no time limit for the participants to respond. The contrast of the target was determined by an adaptive 2-up-1-down staircase procedure: if the participant answered incorrectly, the contrast of the target was increased for the next trial. If the participant answered correctly twice in a row, the contrast of the target was decreased for the next trial. After a total of 60 trials, the responses were fitted with a cumulative Gaussian function. The contrast of the target was then determined by 1.5 times the mean of the fitted cumulative Gaussian. With this procedure, we aimed to find a contrast at which the target can only be detected near the fovea (Bertera & Rayner, 2000).

### Procedure

Each trial contained two search stimuli (Figure 1A). The participants could trigger the onset of the first search stimulus by pressing the space bar while fixating on a white plus to perform a drift check. After 800 ms, the first search stimulus disappeared, followed by a black plus lasting for 1500 ms. After that, the second search stimulus was displayed automatically and lasted for 800 ms. After the second search stimulus disappeared, participants had to answer three questions: (1) Was the target present in the first stimulus? (Yes/No) (2) Was the target present in the second stimulus? (Yes/No) (3) For which stimulus are you more confident about your perceptual judgment? (First/Second). Hence participants were probed about their relative confidence for the two type 1 judgments about the presence/absence of the target in the two intervals. Answers for the first two questions were made by pressing “Up” (Yes) or “Down” (No). The answer for the third question was responded by pressing “Left” (First) or “Right” (Second). For all three questions, there was no time limit for the participants to respond.

### Data analysis

Fixations were detected using the standard Eyelink algorithm. Fixations with a duration of less than 150 ms were excluded (on average 16.65%), assuming that they were not sufficiently long to gather information. We excluded trials when there was no fixation on the search stimulus detected during the search interval or when the participant blinked during the search interval (on average 4.27%).

For each target-present stimulus, we calculated the minimum gaze-target distance as the minimum Euclidean distance between the target location and all fixation locations $\bar{z}$: where *N* indicates the total count of fixations, *z_t_* = (*x_t_, y_t_*) indicates the location of the target, and ${\bar{z}}_{i}=\left({\bar{x}}_{i},{\bar{y}}_{i}\right)$ indicates the average eye position during the *i*th fixation:

(1) $$\underset{i}{\text{min}}z_{t}-{\bar{z}}_{i},1\leq i\leq N$$

We decided to use the minimum distance across all fixations and not just the distance at the last fixation because of several reasons: First, a metric based on one fixation is more prone to noise than a metric based on all fixations in a trial. Therefore, the minimum distance should be a more robust measure. Second, the distance at the last fixation could be larger than the minimum distance for different reasons: on the one hand, participants might have overlooked the target initially and continued searching or on the other hand they might have localized the target and moved back towards the screen centre in anticipation of the end of the trial. This ambiguity of the interpretation of the last fixation can be avoided by using the minimum distance across all fixations.

We calculated the hit rate *HR*, and the false-alarm rate *FAR* as the following probabilities of responses *R* and targets *T*, where the superscripts ^+^ and ^−^ denote presence and absence, respectively:

(2) $$HR=P\left(R^{+}|T^{+}\right)$$

(3) $$FAR=P\left(R^{+}|T^{-}\right)$$

We also calculated the sensitivity d' and the criterion c (Hautus et al., 2021) based on the hit rate *HR* and the false-alarm rate *FAR*:

(4) $$d^{\prime }=z(HR)-z(FAR)$$

(5) $$c=\frac{z(HR)+z(FAR)}{-2}$$

Before calculating d' and c, extreme hit- and false-alarm rates of zero or unity were corrected by adding or subtracting 0.5/N, respectively (Macmillan & Kaplan, 1985).

The influence of scale on search performance was assessed with linear regressions of hit rate, false-alarm rate, d' and c on the search scale. Since quantifying the intercept at a search scale of zero would be meaningless, we subtracted the standard scale of 0.7 before calculating the regression to obtain intercepts at the standard scale. The influence of scale on confidence was assessed with linear regressions of the proportion of selections on the scale difference between the two stimuli.

To assess the influence of the minimum gaze-target distance on the chance to detect the target, we calculated two distributions of distances separately for hits and misses and analyzed the difference between these distributions with an ROC analysis (Hanley & McNeil, 1982). The same procedure was used to analyze the influence of the minimum gaze-target distance on the confidence selection.

To assess the effect of the minimum gaze-target distance in more detail, a sliding window was applied on the distance, with a window size and a step size of 0.5°. The hit rate or the rate of confidence selections was calculated for each window.

In all analyses, participants with less than 6 observations per data point were excluded from that particular analysis.

The alpha-level for statistical tests was set to 5%.

## Results

### Visual search performance

We analyzed search performance by calculating the hit rate, false-alarm rate (Equations (2)–(3)) and sensitivity d' and the criterion c (Equations (4)–(5)) from target-absent vs. target-present responses in the type 1 task. For each participant, linear regressions were fitted as a function of the search scale and aligned relative to the standard scale of 0.7.

**Figure 2:**
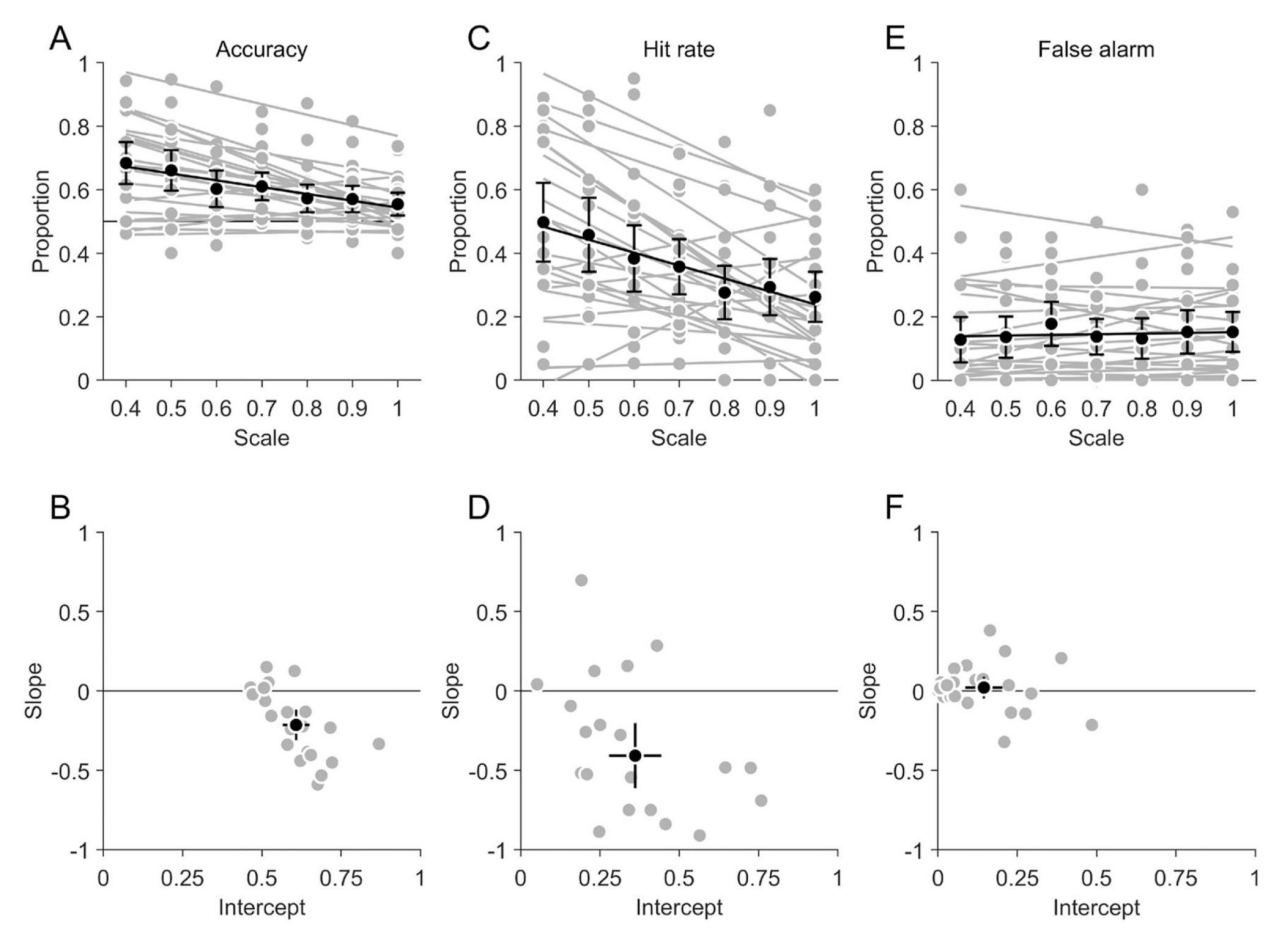
Visual search performance. (A) Accuracy as a function of search scale. (B) Slopes and intercepts of the regression in (A), relative to the standard scale of 0.7. (C) Hit rate as a function of search scale. (D) Slopes and intercepts of the regressions in (C), relative to the standard scale of 0.7. (E) False-alarm rate as a function of search scale. (F) Slopes and intercepts of the regressions in (E), relative to the standard scale of 0.7. (A–F) Grey data points indicate individual participants; black data points the average across participants. Error bars indicate 95% confidence intervals. (A & C & E) Thick lines represent linear regressions.

The regression of accuracy (Figure 2A,B) had a significantly negative slope of −0.21 (95% confidence interval: −0.31, −0.12, t(21) = 4.63, *p* < 0.001), indicating that the accuracy declined with increasing scale. The regression intercept at the standard scale of 0.7 was 0.61 (0.57, 0.65), significantly larger than 0.5 (t(21) = 29.69, *p* < 0.001), which indicates that the participants were able to perform better than chance.

The regression of hit rate (Figure 2C,D) had a significantly negative slope of −0.41 (−0.61, −0.20, t(21) = −4.14, *p* < 0.001), which indicates that hit rate declined with increasing scale. The regression intercept at the standard scale of 0.7 was with 0.36 (0.28, 0.44) significantly larger than zero (t(21) = 9.09, *p* < 0.001), which indicates that the task was difficult but not impossible.

The regression slope of false-alarm rate (Figure 2E, F) was with 0.02 (−0.05, 0.09) not significantly different from zero (t(21) = 0.64, *p* = 0.528), which indicates that the size of the search area did not affect the rate of false alarms. The regression intercept at the standard scale of 0.7 was with 0.14 (0.09, 0.20) significantly larger than zero (t(21) = 5.14, *p* < 0.001), which indicates that participants produced false alarms at a small but significant rate.

The regression of d' (Figure S2A&B) had a significantly negative slope of −1.39 (−2.00, −0.77, t(21) = −4.71, *p* < 0.001), which indicates that search performance declined with increasing scale. The regression intercept at the standard scale of 0.7 was with 0.81 (0.49, 1.12) significantly larger than zero (t(21) = 5.33, *p* < 0.001), which indicates that the task was difficult but not impossible.

To quantify if participants preferred target-absent or target-present responses under uncertainty, we calculated their response criterion c (Figure S2C&D). A positive criterion indicates a conservative response bias with a preference for target-absent responses. The regression intercept was significantly larger than zero at the standard scale of 0.7: 0.83 (0.63, 1.04, t(21) = 8.42, *p* < 0.001), which indicates that participants had a conservative response bias favouring target-absent responses. The regression slope was significantly positive: 0.55 (0.15, 0.94, t(21) = 2.85, *p* = 0.010), which indicates that the response criterion was more conservative at larger scales.

In sum, given the limited search duration, the small number of fixations (see below) and the decrease of search performance with increasing size of the search area, the search was incomplete (at least) at larger sizes and participants used a conservative response criterion.

### Eye movement behaviour and visual search performance

**Figure 3:**
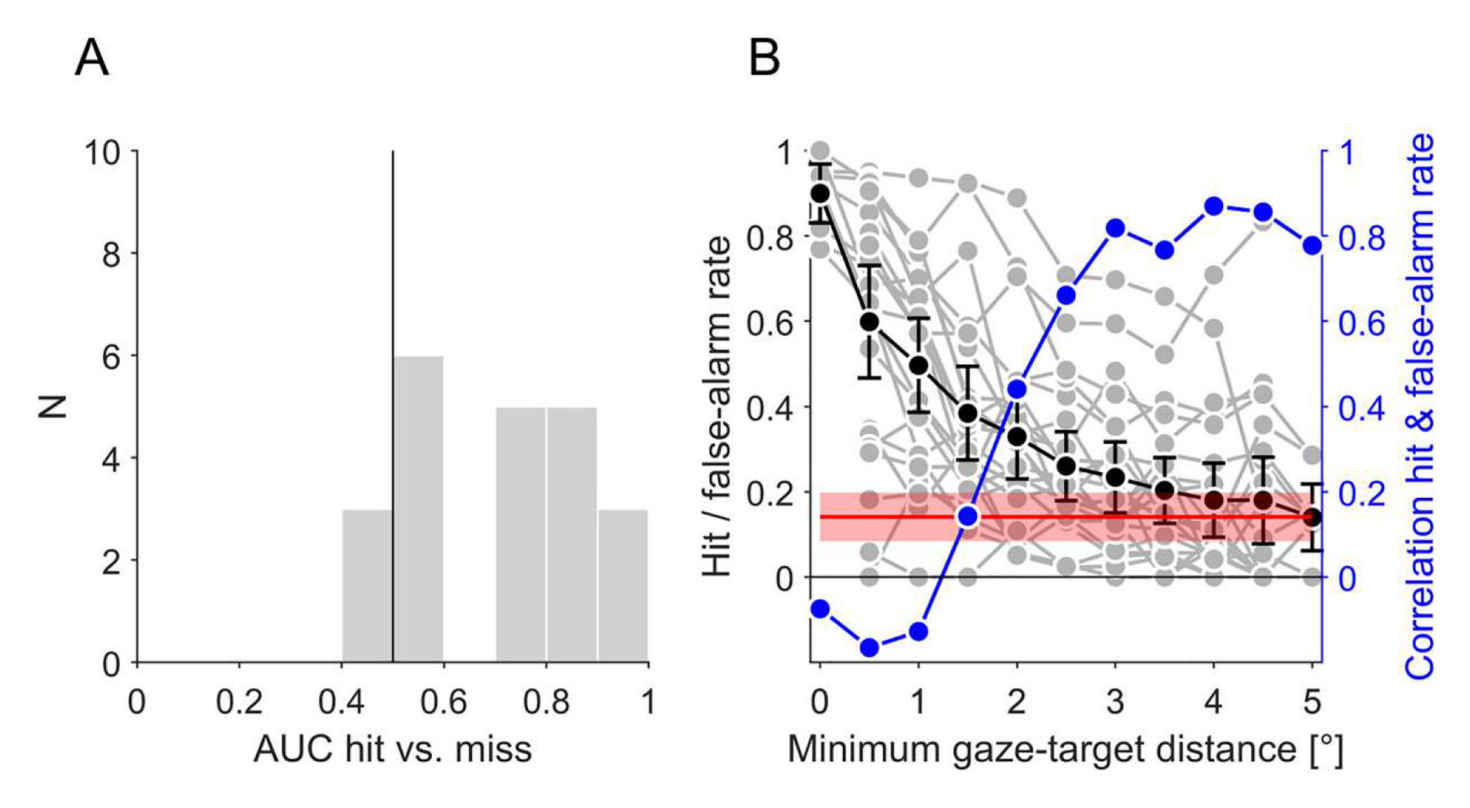
Eye movement behaviour and visual search performance. (A) Area under the curve (AUC) of the minimum gaze-target distance for hit- and miss-stimuli. (B) Hit and false-alarm rate. The hit rate is plotted in black as a function of the minimum gaze-target distance. Grey data points indicate individual participants; black data points the average across participants. Error bars indicate 95% confidence intervals. The horizontal red line indicates the average false-alarm rate; the red shaded area its 95% confidence interval. The blue data indicate the correlation between hit and false-alarm rate across participants for different gaze-target distances.

Participants made on average 2.36 fixations (minimum 1.40, maximum 2.74) during the 800 ms search interval. We can expect that participants could detect the target only if they were fixating close enough to the target location, given the small size and low contrast of the target and the decline of acuity and spatial contrast sensitivity with eccentricity from the fovea (for reviews see Stewart et al., 2020; Strasburger et al., 2011). To test this hypothesis, we calculated (only in target-present stimuli) the minimum distance between the target location and all fixation locations (i.e., the minimum gaze-target distance) in one stimulus. Across all stimuli, we obtained two separate distributions for hits and misses and compared these distributions with an ROC analysis to test if target detection can be predicted by the minimum gaze-target distance. The area under the curve (AUC, Figure 3A) was on average 0.70 (0.63, 0.78) and significantly larger than chance discrimination of 0.5 (t(21) = 5.53, *p* < 0.001). On an individual level, the AUC was larger than chance for 19 of 22 participants. This means that the minimum gaze-target distance was larger for misses than for hits and that it can reliably predict if a participant detected or missed a target.

We can assume that participants cannot accurately detect the target if the gaze-target distance exceeds a certain threshold. Hits beyond that threshold can be considered as lucky guesses. In this case, the asymptotic hit rate for large distances should correspond to the false alarm rate of the participant. To analyze the relationship between target detection and minimum gaze-target distance in more detail, we calculated the hit rate as a function of distance (Figure 3B). The hit rate was largest for the smallest distances and decreased for larger distances. It reached the average false-alarm rate of 0.14 (0.08, 0.20) at a minimum gaze-target distance of about 2°. Given that there was considerable individual variation in hit rates and false-alarm rates, we can calculate the correlation between the false-alarm rate and the hit rate across participants for different gaze-target distances. The correlation was negative for small distances, indicating that participants who could identify the target better when they fixated closely also had a lower false-alarm rate. At larger distances, the correlation was positive and increased until it plateaued at values of around 0.85 for distances larger 3°.

Overall, these results indicate that participants could reliably detect the target only when the gaze location came closer to the target than about 2-3°. Above that threshold, hits were not more frequent than false alarms and hit and false-alarm rates were correlated. This means that eye movements and fixation locations can be used to distinguish true hits from false positives.

### Confidence choices

**Figure 4:**
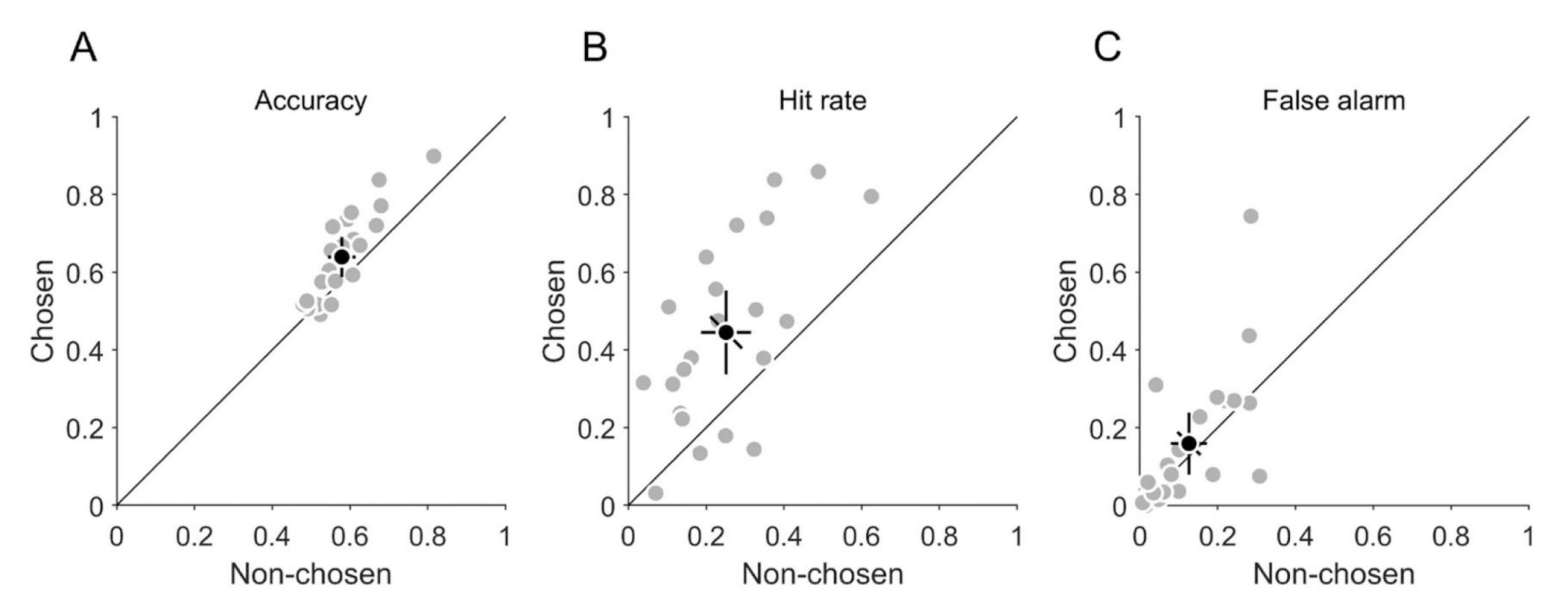
Confidence choices and objective performance. Values are shown for non-chosen and chosen stimuli. (A) Accuracy. (B) Hit rate. (C) False-alarm rate. (A–C) Grey data points indicate individual participants; black data points the average across participants. Error bars indicate 95% confidence intervals. The diagonal error bar indicates the confidence interval of the pairwise difference between x- and y-values and has to be compared to the identity line (Schütz & Gegenfurtner, 2025).

In each trial, participants searched two stimuli and had to choose for which stimulus they were more confident about their target-present vs. target-absent decision. First, we analyzed how search performance differed in chosen and non-chosen stimuli. If confidence choices are sensitive to the objective performance of participants, their search performance should be higher in chosen than in non-chosen stimuli. Indeed, accuracy (Figure 4A) was significantly higher (t(21) = −4.76, *p* < 0.001) in chosen (0.64 [0.59, 0.69]) than in non-chosen (0.58 [0.54, 0.61]) stimuli, and hit rate (Figure 4B) was also significantly higher (t(21) = −4.90, *p* < 0.001) in chosen (0.44 [0.34, 0.55]) than in non-chosen (0.25 [0.19, 0.32]) stimuli. The higher accuracy and hit rate was not achieved by increasing the false-alarm rate (Figure 4C), which was not significantly different (t(21) = −1.16, *p* = 0.259) in chosen (0.16 [0.08, 0.24]) and non-chosen stimuli (0.13 [0.08, 0.17]). Consequently, d' (Figure S3A) was significantly higher (t(21) = −6.23, *p* < 0.001) in chosen (1.11 [0.71, 1.50]) than in non-chosen (0.60 [0.33, 0.87]) stimuli. The criterion (Figure S3B) was significantly lower (t(21) = 2.83, *p* = 0.010) in chosen (0.73 [0.45, 1.02]) than in non-chosen (1.04 [0.84, 1.25]) stimuli. This means that the search performance was better and the decision criterion was less biased in chosen compared to non-chosen stimuli. Hence, participants' confidence choices were sensitive to their objective performance.

**Figure 5:**
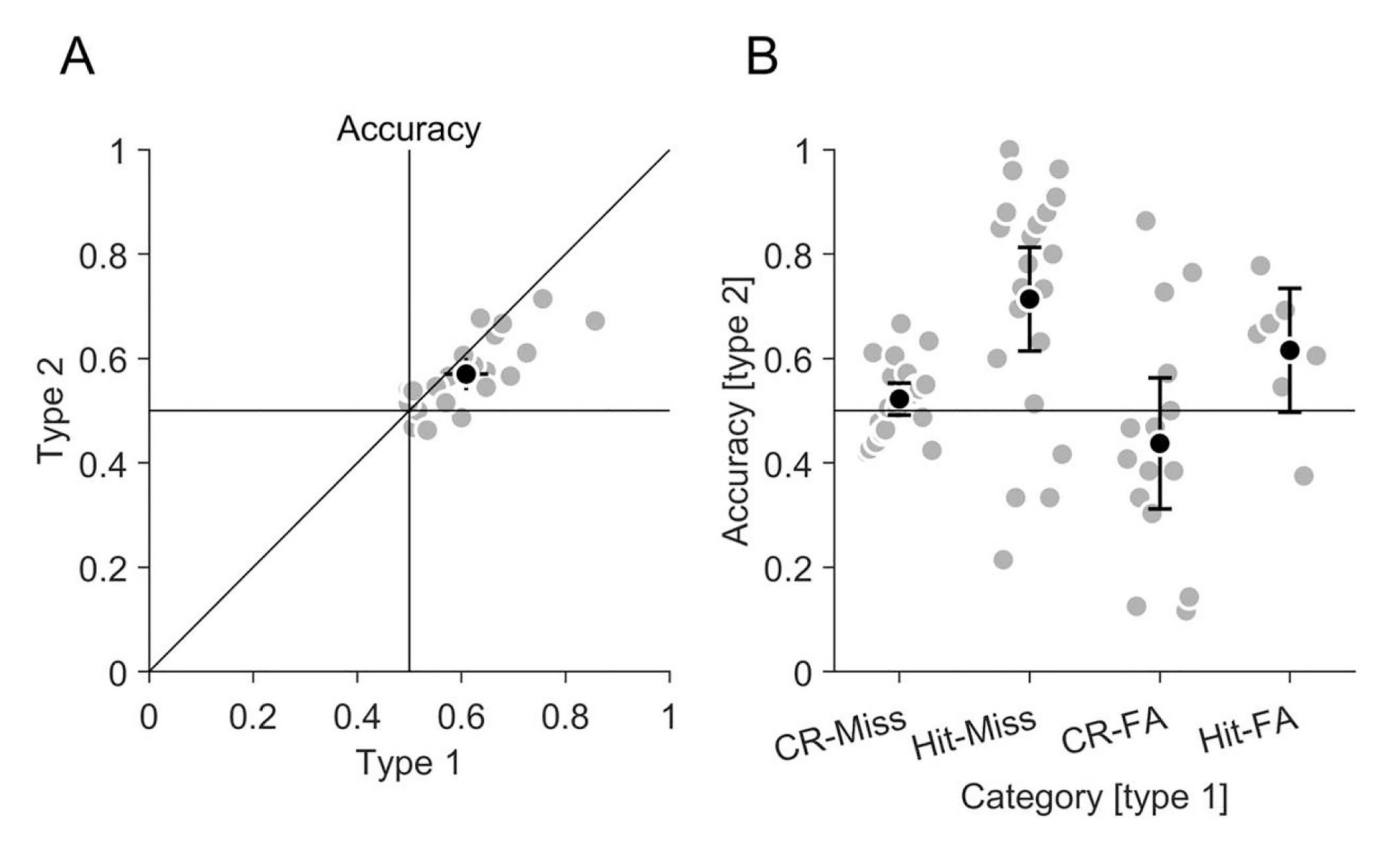
Confidence choices and objective performance. (A) Type 1 and type 2 accuracy. (B) Type 2 accuracy for four combination of type 1 outcomes: correct rejection (CR) & Miss, Hit & Miss, CR & false alarm (FA), Hit & FA. (A & B) Grey data points indicate individual participants; black data points the average across participants. Error bars indicate 95% confidence intervals.

Since we used a confidence forced-choice paradigm, we could assess the objective accuracy of the confidence choice in trials where the type 1 decision was correct for one search stimulus and incorrect for the other search stimulus (proportion of trials: 0.50 [0.45, 0.55]). We compared this accuracy in the type 2 task with the accuracy in the type 1 task (Figure 5A). The type 2 accuracy was with 0.57 (0.54, 0.60) slightly smaller (t(21) = 3.00, *p* = 0.007) than the type 1 accuracy with 0.61 (0.57, 0.65), but both accuracies were correlated (r(20) = 0.77, *p* < 0.001). This indicates that participants performed almost as accurate in the type 2 confidence forced-choice task as their visual search performance in the type 1 task allowed them. To investigate more closely which categories of type 1 decisions could be discriminated well, we further divided the trials according to the combination of outcomes for the two search stimuli (hit, miss, correct rejection and false alarm; Figure 5B). For the discrimination between correct rejections and misses, the accuracy in the type 2 task was with 0.52 (0.49, 0.55) not significantly larger than chance performance of 0.5 (t(21) = 1.50, *p* = 0.149). For the discrimination between hits and misses, the type 2 accuracy was with 0.71 (0.61, 0.81) significantly larger than chance (t(21) = 4.47, *p* < 0.001). For the discrimination between correct rejections and false alarms, the accuracy in the type 2 task was with 0.44 (0.31, 0.56) not significantly larger than chance (t(14) = −1.07, *p* = 0.302). For the discrimination between hits and false alarms, the accuracy in the type 2 task was with 0.62 (0.50, 0.73) trending to be larger than chance (t(6) = 2.39, *p* = 0.054). These results indicate that participants could reliably distinguish hits from misses and from false alarms. Correct rejections however could not be discriminated from misses and false alarms.

**Figure 6:**
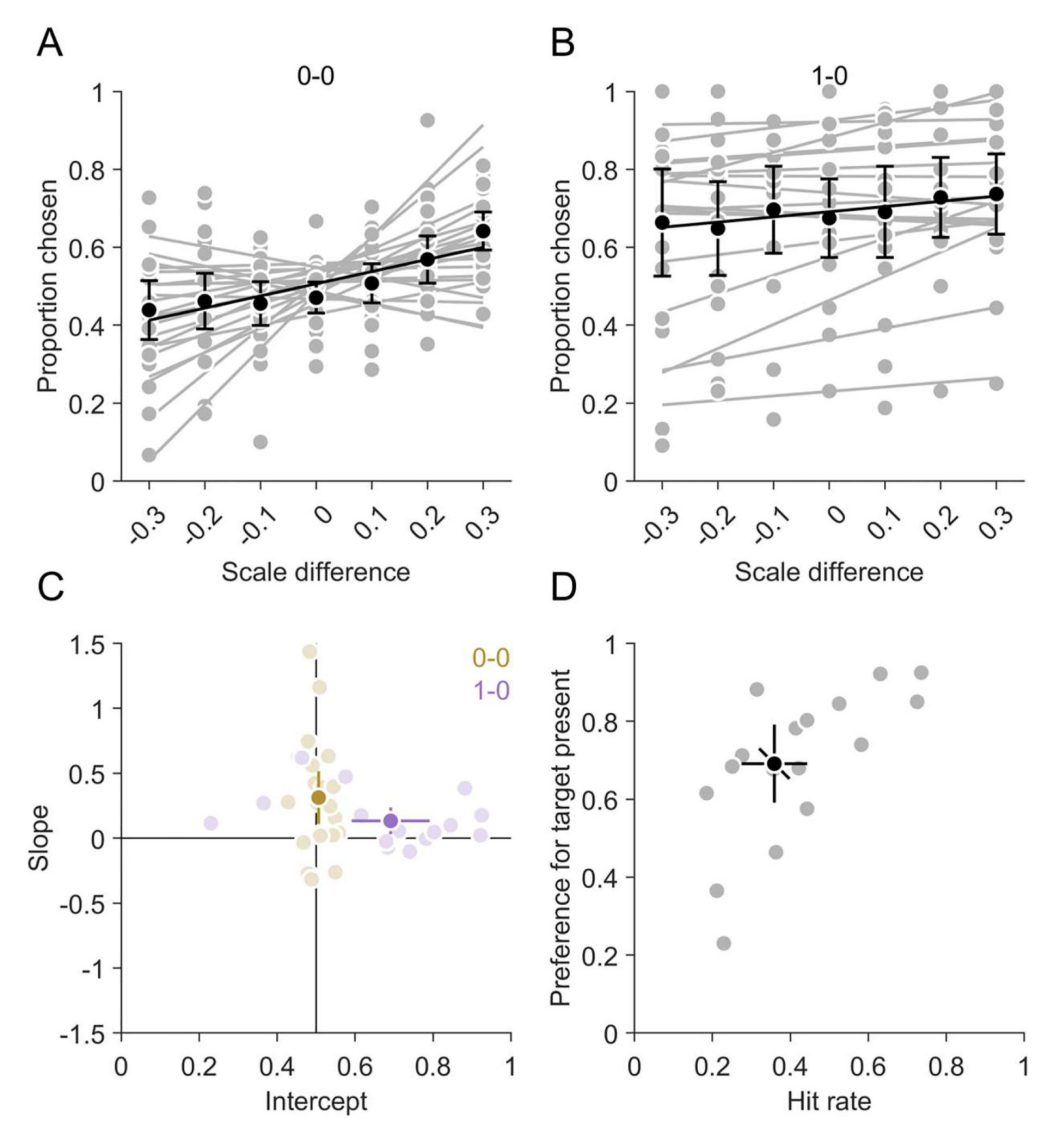
Confidence as a function of scale and target present/absent responses. (A&B) Confidence choices on scale differences between the two stimuli in one trial. (C) Intercepts and slopes of regressions of confidence choices on scale differences between the two stimuli in one trial. The different colours represent different response conditions. (A–C) The first and second digit indicate the target present/absent response in the two stimuli, respectively. (D) Proportion of target-present responses preferred (from C) as a function of the average hit rate.

In the next step, we analyzed which features of the stimulus and the responses influenced the confidence choices. Confidence choices might depend on the scale of the stimuli and the participants' responses about whether the target was absent or present (Reyes & Sackur, 2014). We therefore sorted trials in two conditions according to the target-present vs. target-absent decisions for the standard and the comparison stimulus. For each participant, the proportion of choices for the comparison stimulus were fitted with linear regressions as a function of the difference in the scale between the standard and the comparison stimulus (Figure 6). The slopes can be interpreted as the influence of the scale on confidence choices. When participants gave two target absent responses in a trial (0-0), the slope was with 0.31 (0.11, 0.52) significantly larger than zero (t(20) = 3.20, *p* = 0.004). Also when participants gave a target present response for one stimulus, but not for the other stimulus (1-0), the slope was with 0.13 (0.03, 0.24) significantly larger than zero (t(16) = 2.73, *p* = 0.015). These results indicate that participants were more confident for the smaller stimulus. This is a sensible strategy given that the hit rate was higher (and therefore the miss rate smaller) for smaller stimuli in most of the participants. The intercepts in the regressions of confidence on scale differences can be interpreted as the overall preference for one of the stimuli. When participants gave the same target absent (0-0) response for both stimuli, there was no overall preference as the intercepts were with 0.51 (0.49, 0.52) not significantly different from 0.5 (t(20) = 0.84, *p* = 0.410). However, when the target was reported present in one but not in the other stimulus (1-0), the intercept was with 0.69 (0.59, 0.79) significantly larger than 0.5 (t(16) = 4.05, *p* = 0.001), indicating that the stimulus with the target-present response was chosen more often. Hence, participants were more confident for target-present than for target-absent responses. This is also a sensible strategy because search was not completed, such that a target-absent response carried the uncertainty that the target was present and merely not yet found. The preference for the target present stimulus in the confidence choice was correlated with the hit rate in the type 1 task (r(15) = 0.67, *p* = 0.003), indicating that participants with a high hit rate also had a stronger preference for target present responses.

**Figure 7:**
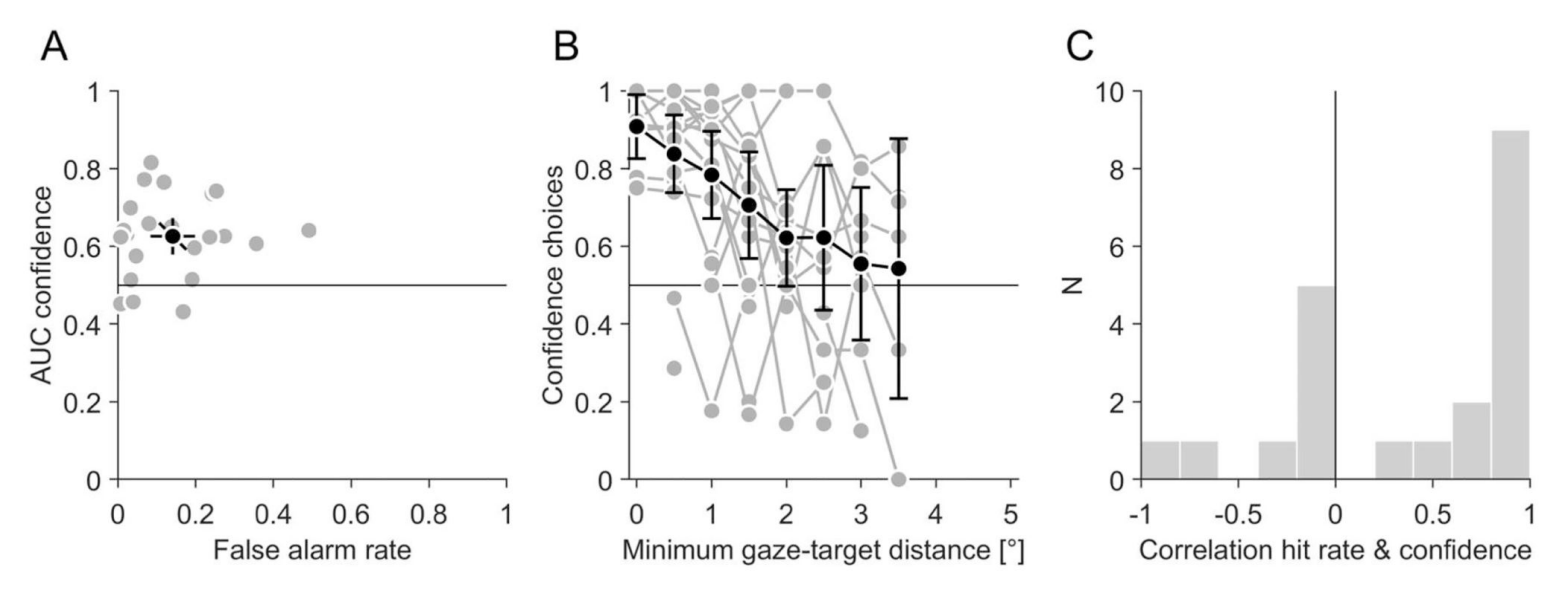
Confidence choices and eye movement behaviour. (A) Area under the curve (AUC) of the minimum gaze-target distance for chosen and non-chosen stimuli in confidence choices as a function of the false alarm rate. Compare to Figure 3A. (B) Confidence choices for the target-present stimulus (when the other stimulus was judged as target-absent) as a function of the minimum gaze-target distance in the target-present stimulus. Compare to Figure 3B. (C) Histogram of correlation coefficients between the hit rate (Figure 3B) and the confidence choices (B).

Finally, we analyzed how confidence choices depended on gaze locations during the search. Since objective performance depended crucially on the minimum gaze-target distance during the search (Figure 3), confidence choices also might be related to this distance. For trials in which a target was correctly detected only in one of the two stimuli, we tested if the minimum gaze-target distance can predict if the stimulus was chosen or not in the confidence task. Across all stimuli, we obtained two separate distributions for chosen and non-chosen stimuli and compared these distributions with an ROC analysis. The area under the curve (AUC, Figure 7A) was on average 0.65 (0.61, 0.69) and significantly larger than chance discrimination of 0.5 (t(21) = 7.23, *p* < 0.001). On an individual level, the AUC was larger than chance for 20 of 22 participants. This means that the minimum gaze-target distance was larger for non-chosen than for chosen stimuli and that it could reliably predict if a participant chose the stimulus or not. This effect of minimum gaze-target distance on confidence choices cannot be explained by a simple high-threshold model (for a review, see Palmer et al., 2000) where participants merely use a binary decision rule for target-present responses to distinguish between true target detections and guesses because even participants with very low false-alarm rates showed this effect (Figure 7A). In other words, even participants who rarely guess with a positive response show an influence of minimum gaze-target distance on confidence choices and are therefore able to discriminate if they got close to the target or not. There was no significant correlation between the false-alarm rate and the AUC (r(20) = 0.07 *p* = 0.769), which also supports the notion that simply distinguishing between true target detections and guesses is not sufficient to explain the confidence choices.

Like for the hit rate, we calculated in more detail how often a stimulus was chosen as a function of the minimum gaze-target distance (Figure 7B). The preference for the stimulus was maximal for the smallest distances, decreased for larger distances and was not significantly larger than chance performance of 0.5 for minimum gaze-target distances of 2° and above. Hence, confidence was not higher for target-present than target-absent responses in general, but only when the minimum gaze-target distance was small. The relationship with distance was quite similar for the hit rate and the confidence choices (Figure 7C): the correlation was on average 0.38 (0.11, 0.66) and significantly larger than zero (t(20) = 2.92, *p* = 0.008). On an individual level, the correlation was larger than zero for 13 of 22 participants. This suggests that participants' subjective choices closely followed the minimum gaze-target distance, very similar to their objective hit rate.

## Discussion

We asked participants to search for a target in two time-limited intervals, to report the target's presence/absence for each interval and to choose for which decision they felt more confident. Since the target was small and of low contrast, the hit rate decreased with increasing search area and with increasing gaze-target distance. At large gaze-target distances, the hit rate was similar and correlated with the false-alarm rate across participants, indicating that those hits were lucky guesses. Search performance was higher in chosen than in non-chosen stimuli, which demonstrates that participants had some metacognitive sensitivity for their own search performance. The accuracy in the confidence task was almost as high as the accuracy in the search tasks. This was primarily accomplished by correct discrimination of hits vs. misses and false alarms, as correct rejections could not be reliably discriminated from misses and false alarms. Confidence choices showed a preference for target-present compared to target-absent responses, but that preference was contingent on the gaze-target distance, similar as the hit rate. This indicates that participants used the visibility of the target to guide their confidence choices. There was also a confidence preference for smaller search stimuli.

When selecting the search stimulus for which they felt more confident, participants preferred stimuli with target-present over stimuli with target-absent responses (Figure 6C). This is in line with a previous report for higher confidence ratings in target-present than in target-absent trials (Reyes & Sackur, 2014). Given that participants had not enough time to search the whole stimulus exhaustively, this confidence bias can reflect the remaining uncertainty when the target has not been detected in a stimulus, yet. In that sense, the lower confidence for target-absent than target-present responses corresponds to longer reaction times in target-absent than in target-present trials in classical paradigms when search duration is not limited (Wolfe et al., 2005). Furthermore, higher confidence for target-present than target-absent responses has been observed in various visual detection and discrimination paradigms (Kanai et al., 2010; Mazor et al., 2020, 2021). Such a metacognitive asymmetry between target-present and target-absent responses might have contributed to our findings beyond the mere uncertainty of unsearched stimulus space due to the limited search duration.

Importantly, the bias for target-present over target-absent stimuli was not a rigid response heuristic, but contingent on the available evidence for the target-present response. The probability to choose a target-present stimulus as subjectively more confident decreased with increasing minimum gaze-target distance during the search (Figure 7B). The same relationship was observed between the objective hit rate to detect the target and the minimum gaze-target distance (Figure 3B). Hit rate and confidence preferences for target-present stimuli peaked at the smallest distance. At large distances, the hit rate was comparable to the false-alarm rate and there was no confidence preference for target-present responses over target-absent responses. This means that the subjective confidence selection was related to the objective performance and modulated by the actual amount of evidence for the target-present response. Target-detections at large distances therefore can be considered as lucky guesses rather than true target-detections. Participants were aware that they did not truly detect the target. Our results show that the analysis of fixation patterns can help to distinguish true target detections from lucky guesses.

A critical question is the nature of the representation of target visibility that is used for the confidence choices. According to a simple high-threshold model, one could assume a discrete, binary decision variable that distinguishes for target-present responses only between true target detections (high confidence) and guesses (low confidence). In this case, the decay of confidence with increasing gaze-target distance would merely reflect different relative probabilities of true target detections vs. guesses. We argue that low-threshold models, which are grounded in signal-detection theory (Green & Swets, 1966) and which involve a more graded decision variable are more likely because of two reasons. First, it has been shown that many aspects of visual search can be better explained by low-threshold models (Eckstein et al., 2000; Palmer et al., 2000; Verghese, 2001). Amongst others, this includes effects of set-size, target-distractor discriminability and response biases. Second, we found that even participants with very low false-alarm rates, i.e., participants who are rarely guessing in the sense of the high-threshold model, exhibit a relationship between gaze-target distance and confidence choices. This means that these participants have a more nuanced representation of target visibility than the two states of the high-threshold model. Whether the underlying representation is continuous or involves some number of discrete steps will require further research.

The amount of evidence for the target-present response could have been estimated by at least two different cues: An obvious cue is how clearly the target was perceived. Due to the decline of contrast sensitivity and visual acuity towards the periphery (for reviews see Stewart et al., 2020; Strasburger et al., 2011), target visibility decreased towards the periphery as evidenced by the reduction of the hit rate. We therefore can assume that participants could have used a visual cue about how well they perceived the target for their confidence judgment. A second, less obvious cue might be the retinal location of the target. Participants might have followed a prior that they can trust information close to the fovea more than further eccentric information. Indeed, several studies showed that confidence and visibility can be dissociated and that retinal location can influence confidence and perceptual inferences above and beyond simple effects of visibility: For instance, foveal information is preferred over peripheral information even under scotopic viewing conditions when the fovea does not provide veridical information due to the lack of rod photoreceptors (Gloriani & Schütz, 2019). Furthermore, it has been shown that stimuli with high spatial uncertainty are mislocalized towards the fovea (Brenner et al., 2008). Both findings suggest that visual uncertainty is underestimated at the fovea compared to the periphery. However, a simple prior based on retinal eccentricity is unlikely to account for the full effect, because it cannot easily explain the correlation between the objective hit rate and the subjective confidence preference (Figure 7C). Hence, it is more parsimonious to assume that confidence preferences were mainly driven by the target visibility at different eccentricities.

When no target was detected in both stimuli, the probability to choose a stimulus as more confident was inversely related to the size of the search area. Again, two different cues might have driven this relationship: First, participants might have used the perceived size of the search area as a general estimate of the difficulty of that stimulus and the associated probability to miss a target. This cue is easily accessible, but does not consider the individual interaction with the stimulus. Prior studies showed that confidence choices are more closely related to performance than to visual cues that could act as a proxy for performance (Barthelme & Mamassian, 2010). Second, they might have taken their own exploration behaviour into account and estimated how much of the search area they have not sufficiently explored yet. This would have the benefit that it allows for a higher sensitivity to distinguish between more and less efficient searches, but also requires meta-knowledge about the own exploration behaviour. There is mixed evidence in the literature about how well humans can remember their own eye movements. On the one hand, some studies provided evidence that humans cannot accurately report their own fixation locations (Clarke et al., 2017; Kok et al., 2017; Marti et al., 2015; Võ et al., 2016). On the other hand, some studies provided evidence that humans retain information about fixated items (Dickinson & Zelinsky, 2007; Foulsham & Kingstone, 2013). For the purpose of estimating confidence in visual search, it is actually not necessary to remember the exact scan path. An estimation of how much information was gathered about the search area would be sufficient. Previous studies showed that humans are capable of such, more implicit estimations. For instance, when comparing two stimuli to each other, participants spend more time looking at the noisier stimulus (Cassey et al., 2013). Furthermore, when participants have to select one of several objects for a subsequent perceptual task, they prefer objects for which they have gathered more information by fixations (Stewart et al., 2022). Interestingly, human searchers can accurately estimate which proportion of the search area they covered in 2D search stimuli, but strongly overestimate the covered area in 3D volumes (Lago et al., 2021). With the current data we cannot distinguish between the two possible cues because the actual size of the search area and the explored proportion of the search area are confounded. Future studies could dissociate these cues by varying additional factors that influence which proportion of the search area can be explored, for instance the search duration.

Our results show that both the objective performance and the subjective confidence in visual search are critically related to how close the gaze reached the target. This means that the visibility of the target and/or the retinal distance to the fovea are cues for perceptual decision making and metacognitive confidence in visual search. Participants can use these cues effectively to enhance the accuracy of target-present responses when estimating confidence for their decisions. Under some conditions, they can also use other cues, such as the size of the search area to improve the accuracy of target-absent responses.

## Supplementary Material

Supplemental data for this article can be accessed online at https://doi.org/10.1080/13506285.2025.2571626.

Supplementary Material

## Data Availability

Data and analysis code are available at https://doi.org/10.5281/zenodo.17350966.
