# Supplementary material for: Confidence in incomplete visual search
Source: Vis cogn. Author manuscript; Available in PMC 2026 Jan 16. (PMC7618635; doi:10.1080/13506285.2025.2571626)
Supplement: Supplementary Material [file EMS211996-supplement-Supplementary_Material.pdf]

Supplementary material:
Confidence in incomplete visual search

Hui Men[1], Alexander C. Schütz[1,2]

1 Allgemeine und Biologische Psychologie, Philipps-Universität Marburg, Marburg, Germany

2 Center for Mind, Brain and Behaviour, Philipps-Universität Marburg, Marburg, Germany

Analysis of order effects

Search performance and confidence might differ between the first and second interval and therefore add noise to our measurements. For instance, participants might have performed worse in the second interval because they had to remember their decision about the target's presence/absence from the first interval. Alternatively, they might have performed worse in the first interval, because they forgot about their decision until probed after the second interval. To check for possible order effects, we calculated hit rate, false-alarm rate, d' and the criterion, separately for the first and the second interval. The accuracy (Figure S1A) was highly correlated ($r(20) = 0.83$, $p < 0.001$), but not significantly different ($t(21) = -0.49$, $p = 0.626$) in the first (0.61 [0.56, 0.65]) and the second interval (0.61 [0.57, 0.66]). The hit rate (Figure S1B) was highly correlated ($r(20) = 0.91$, $p < 0.001$), but not significantly different ($t(21) = -0.36$, $p = 0.720$) in the first (0.36 [0.27, 0.44]) and the second interval (0.36 [0.28, 0.45]). The false-alarm rate (Figure S1C) was highly correlated ($r(20) = 0.90$, $p < 0.001$), but not significantly different ($t(21) = -0.63$, $p = 0.534$) in the first (0.14 [0.08, 0.19]) and the second interval (0.14 [0.08, 0.21]). The sensitivity d' was highly correlated ($r(20) = 0.89$, $p < 0.001$), but not significantly different ($t(21) = 0.23$, $p = 0.810$) in the first (0.90 [0.57, 1.24]) and the second interval (0.88 [0.52, 1.25]). The criterion c was highly correlated ($r(20) = 0.95$, $p < 0.001$), but not significantly different ($t(21) = 0.90$, $p = 0.379$) in the first (0.87 [0.64, 1.10]) and the second interval (0.84 [0.63, 1.05]). Hence, search performance was not different between the first and second interval, but in fact highly correlated. Therefore, we can conclude that the confidence forced-choice paradigm did not introduce additional noise and that our measurements were highly reliable.

Participants might have been more confident about the second interval, because less time passed compared to the first interval. To test if participants preferred one of the two intervals in the confidence task, we calculated for each participant how often they chose the first interval as more confident (Figure S1D). This proportion was on average 0.49 (0.46, 0.53) and not significantly different from 0.5 ($t(21) = -0.29$, $p = 0.772$). Hence, there was no general preference for one of the two intervals. This was also the case for individual participants, where the proportions ranged from 0.31 to 0.65, such that the maximum absolute deviation from equal selection of the first and second interval was 0.19.

These results overall indicate that the confidence-forced choice paradigm results in highly reliable and unbiased measurements.

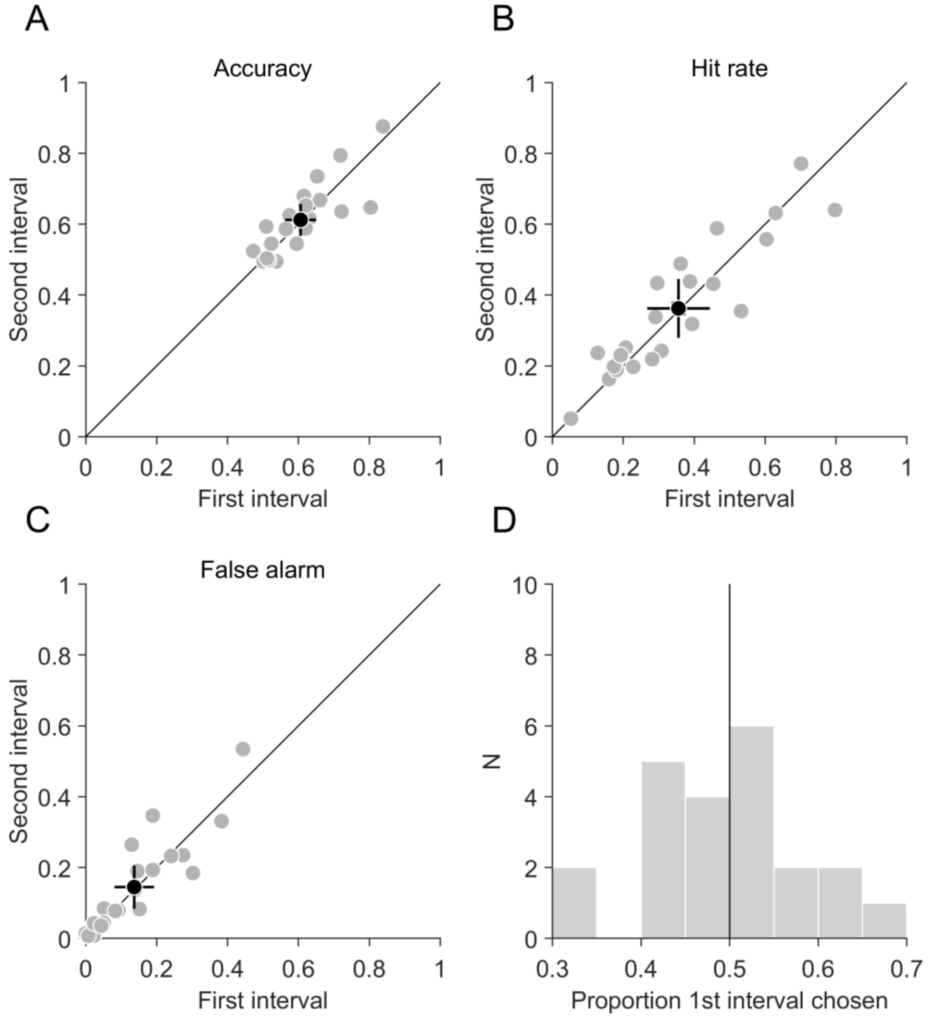

Figure S1: Analysis of order effects. (A) Accuracy for the first and the second interval. (B) Hit rate for the first and the second interval. (C) False-alarm rate for the first and the second interval. (A-C) Gray data points indicate individual participants; black data points the average across participants. Error bars indicate 95% confidence intervals. (D) Histogram of proportion of first interval chosen as more confident.

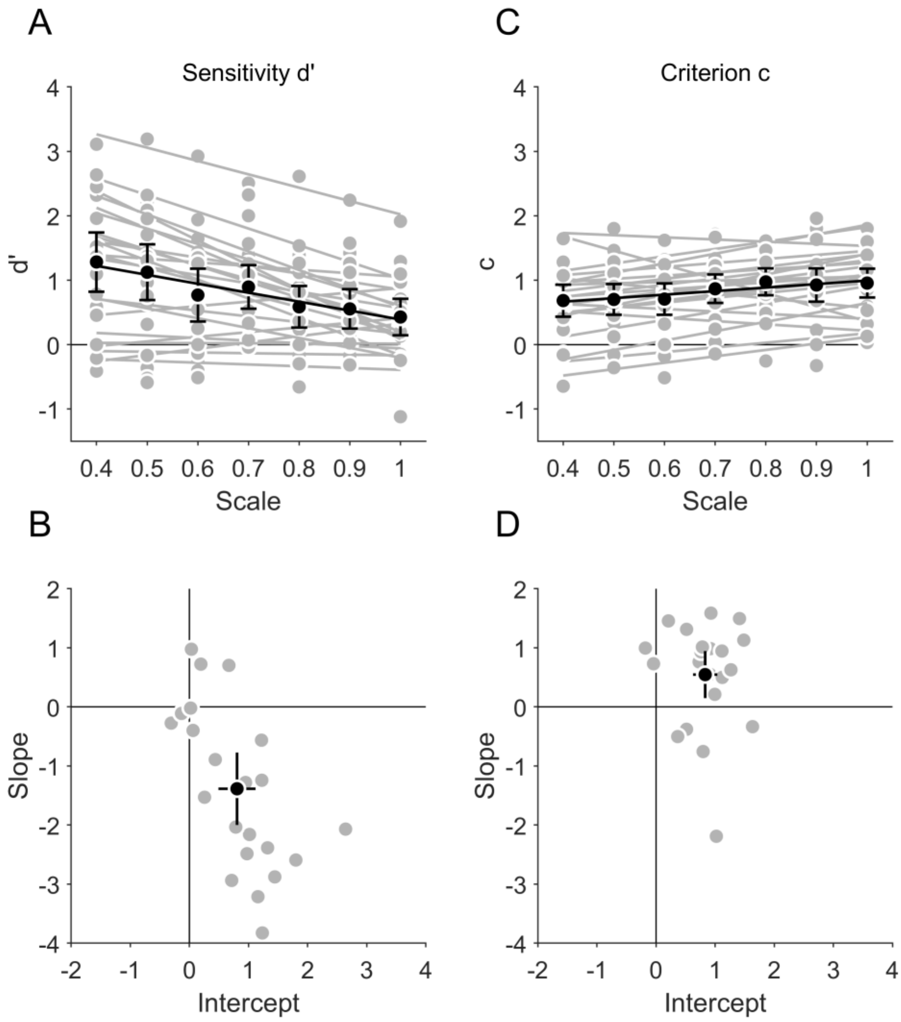

Figure S2: Visual search performance. (A) Sensitivity d' as a function of search scale. (B) Slopes and intercepts of the regressions in (A), relative to the standard scale of 0.7. (C) Criterion c as a function of search scale. (D) Slopes and intercepts of the regressions in (C), relative to the standard scale of 0.7. (A-D) Gray data points indicate individual participants; black data points the average across participants. Error bars indicate 95% confidence intervals. (A & C) Thick lines represent linear regressions.

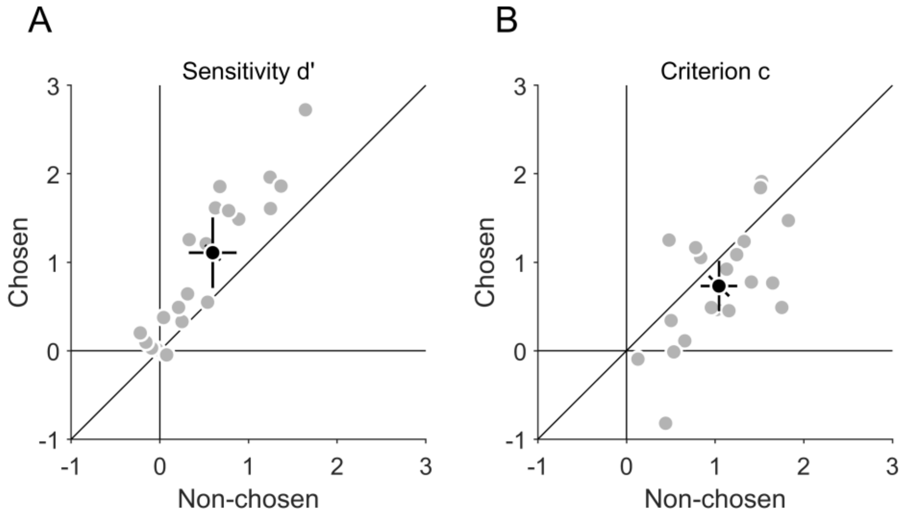

Figure S3: Confidence choices and objective performance. Values are shown for non-chosen and chosen stimuli. (A) Sensitivity d'. (B) Criterion c. (A-B) Gray data points indicate individual participants; black data points the average across participants. Error bars indicate 95% confidence intervals.
